# Cysteine-rich domain of scavenger receptor Al modulates the efficacy of surface targeting and mediates internalization of oligomeric beta amyloid

**DOI:** 10.1186/1750-1326-8-S1-P43

**Published:** 2013-09-13

**Authors:** Huey-Jen Tsay

**Affiliations:** 1 Institute of Neuroscience, National Yang-Ming University, Taipei, Taiwan

## Background

Scavenger receptor class A (SR-A) of microglia and macrophage mediates the internalization of oligomeric amyloid-β peptide (oAβ) and low-density lipoprotein in Alzheimer’s disease and atherosclerosis. SR-A is a member of the cysteine-rich domain (SRCR) superfamily, but the function of the SRCR domain is unclear.

## Materials and methods

We investigated whether the SR-AI SRCR domain encoded by exons 10 and 11 modulates receptor surface targeting, ligand internalization, and extracellular matrix adhesion by expressing mutated SR-A variants in COS-7 cells.

## Results

We found that SR-A variants with truncated exon 11 were intracellularly retained, whereas SR-A variants with further truncation into exon 10 were surface-targeted. Surface-targeted variants were fully glycosylated, whereas intracellularly-retained variants remained in high-mannose states. The fusion of exon 11 with a surface-targeted SR-A variant resulted in intracellular retention and a high-mannose state. Both the SRCR and collagenous domains mediated the ligand binding, but the collagenous domain was more important for matrix adhesion. Point mutations in a long stretch of β sheet 1, 2 and a loop region between β sheet 4 and 5 of the SRCR domain resulted in intracellular retention and a high-mannose state.

## Conclusions

By identifying the function and critical motifs of the SRCR domain, our study suggests possible approaches to modulate innate immunity in Alzheimer’s disease and atherosclerosis.

